# Brain activity during a cognitive task after consuming food of varying palatability

**DOI:** 10.3389/fpsyg.2025.1522812

**Published:** 2025-04-29

**Authors:** Hongjia Li, Siyao Li, Kenji Matsuo, Tsuyoshi Okamoto

**Affiliations:** ^1^Graduate School of Systems Life Sciences, Kyushu University, Fukuoka, Japan; ^2^Nichirei Foods Inc., Chuo-ku, Tokyo, Japan; ^3^Faculty of Arts and Science, Kyushu University, Fukuoka, Japan

**Keywords:** food deliciousness, EEG, Stroop effect, cognitive task, work efficiency, lateralized brain activity

## Abstract

It has been reported that various physiological and psychological changes occur after consuming delicious food. Additionally, research on task performance efficiency following the consumption of delicious food has garnered significant attention. In particular, studies on physiological states have been actively conducted in recent years, with an increasing number of studies utilizing brain activity measurements. Therefore, in this study, we aimed to investigate the physiological changes that occur after consuming delicious food by administering a cognitive task, the Stroop task, and measuring brain activity during the task. In this study, two experiments were conducted to better understand the effects of consuming delicious food. Before starting the two experiments, we evaluated the taste of fried rice in a preliminary experiment and selected three types of them (delicious, slightly delicious, and normal) for the main experiment. In Experiment 1, 20 healthy students (19–26 years old, 11 females) were divided into two groups: 10 students in group 1 ate delicious fried rice and 10 students in group 2 ate normal fried rice. One experimental block included recording the EEG, performing the Stroop task, eating the given sample, and answering the questionnaire. Results of data analysis indicated that group 1 was significantly shorter (high work efficiency) than group 2 in terms of task work time. Concerning brain activity, group 1 showed lower theta and alpha amplitudes in the frontal regions (high arousal), and alpha band activity was lower in the left frontal region than in the right region (high approach motivation). In Experiment 2, 28 healthy students were asked to eat delicious fried rice and slightly delicious fried rice on different days. The daily experimental flow was set up as in Experiment 1. Results of data analysis showed that deliciousness and EEG were negatively correlated at theta and alpha band. This study provides novel evidence that eating delicious food increases work efficiency, arousal, and motivation for the task and decreases theta and alpha activities in limited brain regions. The observed neural profiles may enhance attentional states in high-demand occupational settings, while providing preliminary insights into neurophysiological mechanisms that might underlie food-related motivational processes.

## Highlights

- EEG recording with cognitive tasks was used to evaluate brain activity after consuming delicious food.
- Eating delicious food decreased EEG alpha amplitudes and increased arousal.
- Eating normal food increased EEG theta amplitudes and caused negative emotions.
- Lateralized activity in frontal brain region reflected approach motivation during the Stroop task.
- Eating delicious food increased work efficiency.

## 1 Introduction

Food deliciousness has been evaluated in many studies for a long time. As there are many deliciousness factors that are difficult to assess directly, most studies have proposed indirect evaluations, such as studying the appearance of food (Spence et al., 2022), watching food advertisements (Egbert et al., 2022), food picture tasks (Kirsten et al., 2022), food classification tasks (Mergelsberg et al., 2019), and so on. Studies have also evaluated deliciousness via subjective evaluation by using cognitive tasks (Giboreau and Meiselman, 2018). Additionally, many studies on deliciousness assessment involved tasks without food intake. However, subjective evaluations cannot be as robust as objective evaluations. Moreover, deliciousness cannot be properly evaluated without directly eating the food. Therefore, we focused on evaluating deliciousness using brain activity, which has become popular in food science in recent years (Semenova et al., 2023). However, directly assessing the perception of taste using brain activity remains a significant challenge, especially for EEG. Previous research on EEG has shown that alpha waves increase and FAA (frontal alpha asymmetry) becomes positive value when people feel relaxed and positive. Therefore, in this study, we hypothesize that EEG data recorded after the consumption of palatable food may indirectly reflect enhanced approach motivation and a heightened state of arousal. At the same time, behavioral data may demonstrate more sequential and accurate responses to the tasks, indicating improved cognitive and motor coordination. To explore this possibility, we conducted an investigation into brain activity following the consumption of delicious food.

Fried rice is a starchy food item that is based on rice and is a traditional staple food in Asia. It provides the energy needed for human activities and is widely consumed by consumers in their daily lives due to its soft and delicate taste (Long et al., 2024). Furthermore, the consumption and purchase of frozen fried rice have increased in recent years due to the need for convenience. Therefore, for this study, we selected a staple food that is commonly eaten in daily life as our initial focus.

### 1.1 Stroop effect

The Stroop task is widely used to evaluate attention, concentration, and cognitive processing. The Stroop effect is a phenomenon in which two types of information presented simultaneously causing cognitive overload. The frequently used stimuli are words of the same color and words of different colors. In recent years, an increasing number of studies have been conducted on task switching, cue switching, and food evaluation using the Stroop task, in which the Stroop effect has been observed (Liu et al., 2022). For example, a study on vegetables using the emotional Stroop task found that attention was particularly focused on vegetables with undesirable sensory characteristics, suggesting that attention plays a role in vegetable acceptability (Agovi et al., 2022). A study on coffee, conducted in parallel with the Stroop color-word task to assess its effect on executive function demonstrated improved planning, creative thinking, and event-, time-, and action-based prospective memory (Soar et al., 2016).

### 1.2 Electroencephalogram

Electroencephalography (EEG) is extensively used to examine brain activity. EEG components, such as theta, alpha, beta, and gamma waves, can reflect various physiological and psychophysical states. Many studies have shown that theta waves appear during discomfort, pleasure, and sleepiness, while one is concentrating on something (Tan et al., 2024) or while retrieving episodic memory (Roberts et al., 2018); alpha waves appear during the resting state with eyes closed; beta waves appear when one is awake, alert, attentive to surrounding events, and is thinking (Tempel et al., 2020); and gamma waves appear during nervousness and attention (Spencer, 2009). Among these, theta and alpha waves are often used to study food. Theta waves have been extensively studied in cognitive tasks related to episodic memory. Specifically, in a study using a word-memory task, participants learned words and were tested on their word memory. The results showed a direct relationship between theta activity and episodic memory retrieval, indicating that theta activity enhances memory performance (Roberts et al., 2018). Previous studies have demonstrated that alpha-band oscillations exhibit both sustained and transient amplitude enhancements contingent upon the presentation of task-relevant stimuli within experimental paradigms (Başar, 2012).

Alpha waves represent the participants’ motivation toward food. A study examining the differences in cerebral hemispheric activation associated with different hedonic responses to a single compound at low concentrations showed a decreasing trend in alpha associated with the liking response (Owen and Patterson, 2002). A study on the hedonic valuation of food using self-reported items from the hedonic attitude to food and the behavioral activation system also showed that relatively dominant left-to-right-sided alpha in the parietal regions of the brain during open-eye recording was associated with higher scores in the hedonic valuation of food (van Bochove et al., 2016). They found that alpha asymmetries were important in the study of food. A more detailed analysis suggested that left frontal alpha asymmetry (FAA) is closely related to approach motivation. In a study on accepted (liked) and non-accepted (disliked) drinks, heart rate and EEG were measured during the tasting of personally selected beverages. Although there were no significant results for FAA, it was claimed to be noteworthy (Lagast et al., 2020). Therefore, in this study, we focused on the left frontal alpha.

### 1.3 Brain regions on cognition

Several brain regions are involved in cognitive task performance. The somatosensory cortex performs many functions, including tactile perception (Pei et al., 2010), body perception (Michael et al., 2022), and motor control (Lee et al., 2008). Many studies have shown that the somatosensory cortex is also involved in cognitive functions and decision-making (Pleger and Villringer, 2013). Somatosensory factors have been suggested for studying the relationship between taste and food. For example, Green and Nachtigal (2012) conducted a study in which participants were asked to rate the intensity of sweet, sour, salty, bitter, fresh, and savory tastes either (1) immediately after the stimulus without moving the tongue (passive tasting) or (2) after touching the tongue to the palate and swallowing once (active tasting). The results revealed that voluntary movement of the mouth and tongue enhanced savory taste. Studies have also focused on attention in the somatosensory cortex. Time-frequency analyses conducted in healthy adults to investigate the effects of attention on somatosensory oscillations in humans (Dockstader et al., 2010) suggested that later non-phase-locked rhythms reflect somatosensory events that are modulated by selective attention. Consequently, changes in the somatosensory cortex are expected to occur during the task after eating.

In the Stroop task, commonly used in many experiments, stimuli are presented as words, requiring participants to comprehend their meanings. Therefore, the brain regions involved in language are assumed to be active. Three classical language areas are known to be involved in language production (Broca’s area) and processing (Wernicke’s area and the angular gyrus). Broca’s area is a region in the frontal lobe of the dominant hemisphere, usually the left hemisphere, linked to speech production. Wernicke’s area is a region of the superior temporal gyrus in the dominant cerebral hemisphere linked to speech and language understanding. It is also related to the comprehension of written and spoken language. The angular gyrus is a region of the brain lying mainly in the anterolateral region of the parietal lobe. Its important function is to transfer visual information to the Wernicke’s area to create meaning from visually perceived words.

### 1.4 Objectives and summary of results

In this study, we aimed to physiologically evaluate the effect of eating delicious food on EEG using a traditional cognitive task (the Stroop task). After analyzing the working time for the Stroop task in each block, we found there were significant differences between the participants in samples 1 and 2. Regarding the EEG data, the overall alpha amplitudes of group 1 were lower than those of group 2 across all blocks. Furthermore, lateralization analyses showed significant differences between groups 1 and 2 in blocks 1 and 3, respectively.

To the best of our knowledge, this is the first study to combine Stroop tasks and EEG to evaluate food deliciousness immediately after eating.

## 2 Experiment 1

### 2.1 Materials and methods

#### 2.1.1 Participants

Twenty-five students (13 females, 19–26 years old, 1 left-handed, no history of neuropsychiatric illness or hearing impairment, and normal hearing in both ears) participated in this study. Three participants dropped out of the experiment because of poor physical health. Two other participants were excluded from the analysis because of errors in the data due to equipment malfunction. Only 20 participants were included in this experiment. The mean patient age was 23 years (range, 19–28 years, SD: 2.6). All the patients were from Kyushu University, Japan. All the participants had normal or corrected-to-normal vision. They were divided into two groups: 10 students in group 1 ate only sample 1, which was considered more delicious; and the remaining 10 students in group 2 ate only sample 2, which was considered comparatively less delicious. Participants were asked to fast for at least 2 hours before the experiment to minimize individual variability in hunger, which has been shown to modulate pleasantness and intake of palatable foods (Guy-Grand et al., 1994). It was emphasized that they should do so to align hunger conditions across participants.

This experiment started at 10/01/2022, and ended at 07/05/2022. Ethical approval for the involvement of human participants in this study was granted by Kyushu University Research Ethics Committee, Reference number 202104, 9/15/2021. Participants gave informed consent via the statement “I am aware that my responses are confidential, and I agree to participate in this survey” where an affirmative reply was required to enter the survey. They were able to withdraw from the survey at any time without giving a reason. The products tested were safe for consumption.

#### 2.1.2 Food samples

A preliminary experiment was conducted before the start of the experiment. For the preliminary experiment, five commercial frozen fried rice samples were prepared. To select two food samples for our experiment, a preliminary experiment on the taste ratings of five candidates was conducted.

Another 10 participants were recruited for the preliminary experiment (male: 3, females: 7, no history of neuropsychiatric illness or hearing impairment, and normal hearing in both ears). All the participants were from Kyushu University. All the participants had normal or corrected-to-normal vision.

We provided 50 g (±2 g) of each of the five types of fried rice including two diced roasted pork. The heating time was determined according to the cooking instructions described on the packaging. After the participants ate the samples, they were instructed to complete a questionnaire and start rating each fried rice sample. The eating time was not restricted. Participants rinsed their mouths with water after eating one sample.

The fried rice with the highest average score (7.6 out of 10) was selected as sample 1, and the fried rice with the lowest average score (6.3 out of 10) from different manufacturers was selected as sample 2 in this experiment.

#### 2.1.3 Equipment and environment

**Figure 1:**
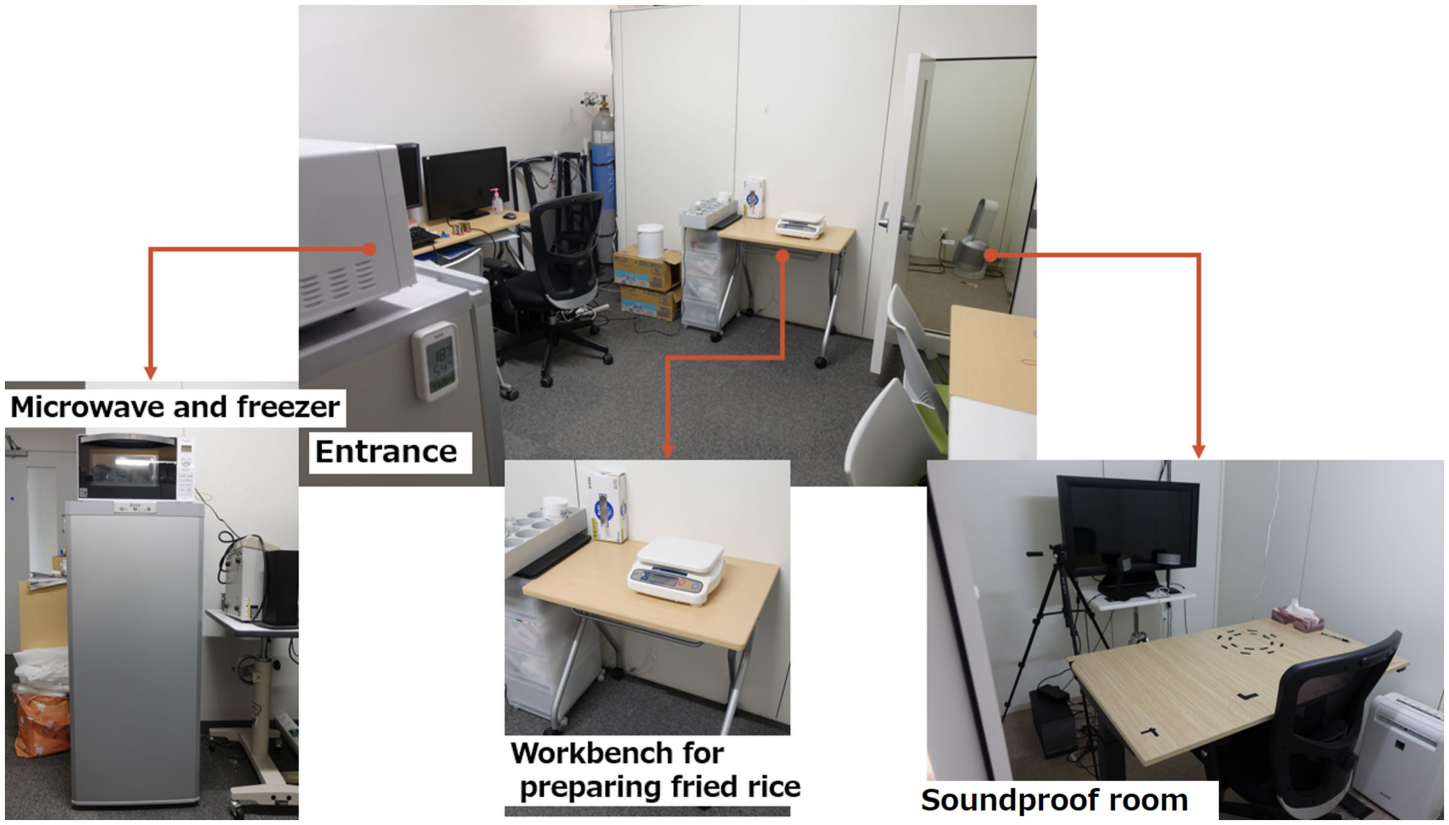
Equipment and environment of the experiment.

The experimental environment is shown in Figure 1.

The laboratory was equipped with a microwave oven (TOSHIBA, Tokyo, Japan), refrigerator (Panasonic, Osaka, Japan), thermal camera (Nippon Avionics Co., Ltd.), weighing scale (A&D Systems, Tokyo, Japan), disposable paper plates, spoons, and drawing paper. When the participants entered the room, fingertip disinfection was performed before the temperature was recorded. Participants were briefed on the experiment and asked to complete a consent form. Food allergies were checked again.

For the fried rice cooking method in this experiment, the fried rice was first removed from the refrigerator and divided into 50 g on small paper plates. Each plate was heated according to the cooking method described on the fried rice packaging. The fried rice was served to participants with surface temperatures aligned around 50°C, as measured by the thermal camera. The working time to cook the fried rice was less than 2 min.

This experiment was conducted in a soundproof room.

#### 2.1.4 Experiment flow

Before the experiment began, the flow of the experiment and the Stroop task were explained to the participant, and the participant was asked to perform a 10-trial practice Stroop task to get accustomed to pressing the response key in the soundproof room with the light off.

**Figure 2:**
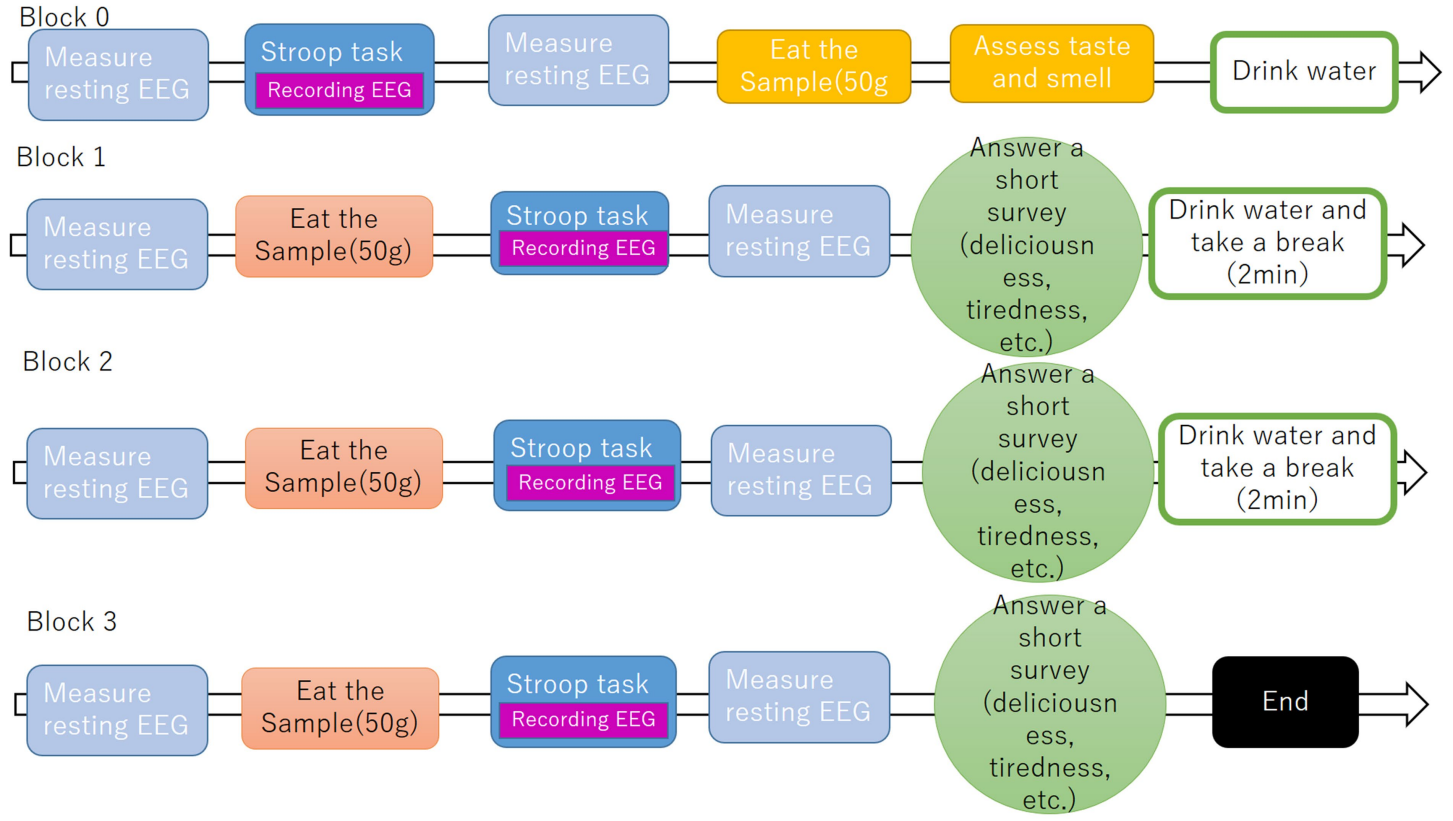
Experiment flow.

To establish baseline measurements and investigate both longitudinal changes in participant responses and differential effects modulated by satiety levels, the experimental protocol was structured into the baseline block and the three experiment blocks. This design enabled systematic isolation of (a) temporal dynamics across repeated measurements and (b) state-dependent variations associated with gastrointestinal satiation states. EEG was recorded while the participants rested with their eyes closed. This experiment consists of four blocks. In block 0, resting EEGs were measured, and the participants were asked to perform a cognition task called the Stroop task to familiarize themselves with the procedure of the task and the experiment. In block 1, resting EEGs were first measured, and each participant was then asked to eat 50 g of the assigned fried rice. After eating, participants were asked to perform a Stroop task. After the Stroop task, resting EEGs signals were measured and the participants were asked to answer a short survey about their current statement. To avoid affecting the next block, the participants were asked to drink water and take a 2-min break to eliminate odor and flavor in the mouth. Blocks 2 and 3 were performed using the same procedure as block 1 (Figure 2).

Across all blocks, the light in the soundproof room was turned off during EEG measurements, and when participants began eating samples or answering questionnaires, the light was turned on.

This experiment took approximately an hour and was completed at the end of block 3.

#### 2.1.5 Questionnaire

There were two types of questionnaires in this study. One was used in block 0, which was designed to confirm the participants’ evaluation of the taste of fried rice. In this questionnaire, participants were asked to evaluate the taste and smell of fried rice. In addition, participants were asked to provide overall ratings of the deliciousness of the fried rice they have eaten.

The other was used in blocks 1, 2, and 3 and was used to confirm the participants’ current state. After the current block was completed, the participants were asked to indicate their states, including current satiety, fatigue, and deliciousness. This was used as a reference for subsequent analyses.

#### 2.1.6 Stroop task

**Figure 3:**
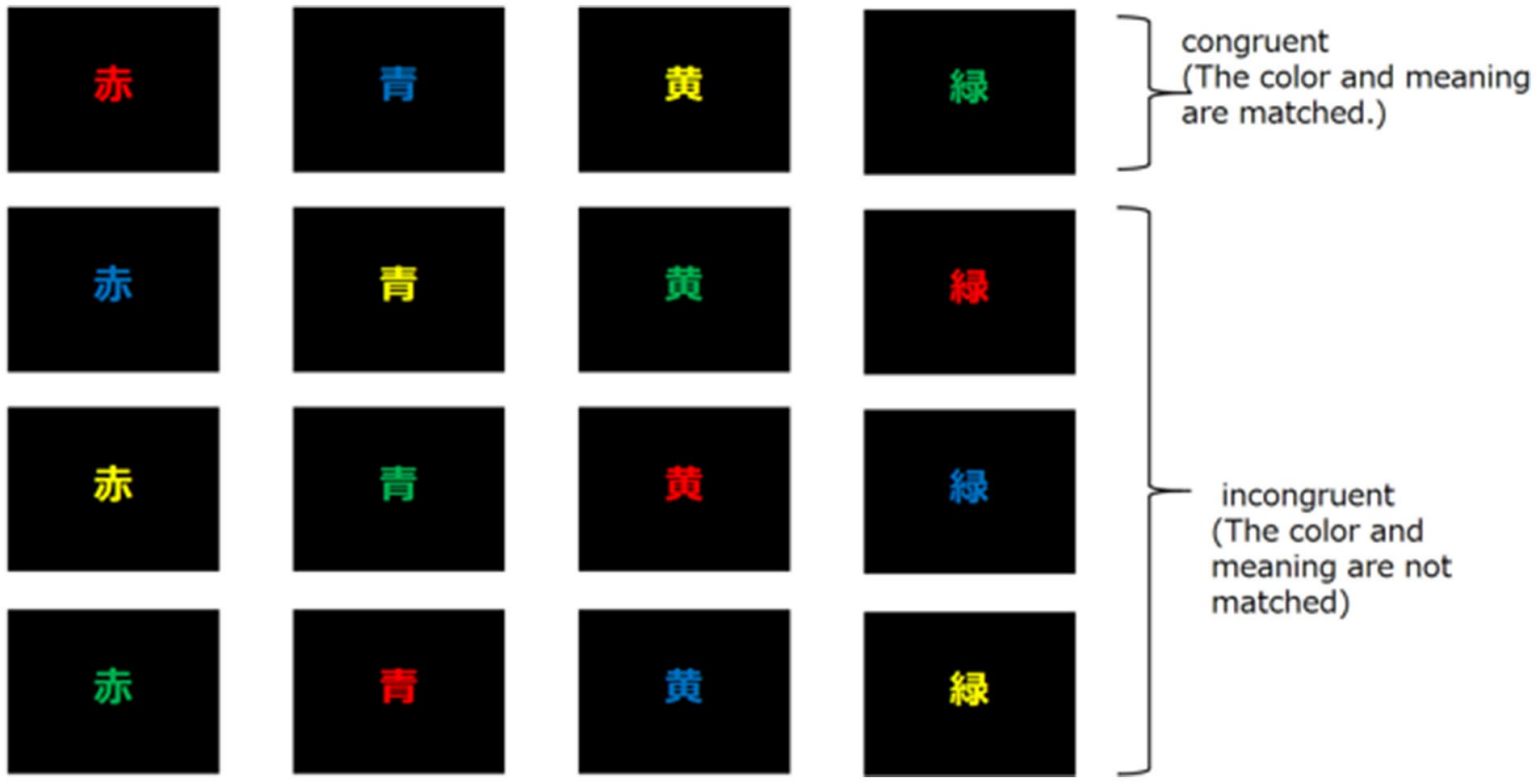
Stimulus of the Stroop task. Two stimulus sets used in the experiment are shown. In this experiment, one of 16 stimuli was selected randomly and presented in one trial.

The widely accepted conflict monitoring theory for Stroop task has been used in brain research. In this study, all participants were native speakers of either Chinese or Japanese. Given the already short response time and the large number of trials, we considered that adding further cognitive load might lead to increased fatigue and potentially affect task performance. Therefore, we used kanji characters that were familiar and easily recognizable to all participants, in order to minimize unnecessary processing burden during the task. The stimuli consisted of four different kanji characters, such as “赤,” “黄,” “青,” and “緑.” “赤” means red, “黄” means yellow, “青” means blue, and “緑” means green. Each kanji was shown in a different color. There were two types of stimulus sets. One is called a congruent stimulus, in which the color and meaning of the stimulus match. The other is called an incongruent stimulus, in which the color and meaning of the stimulus do not match (Figure 3).

**Figure 4:**
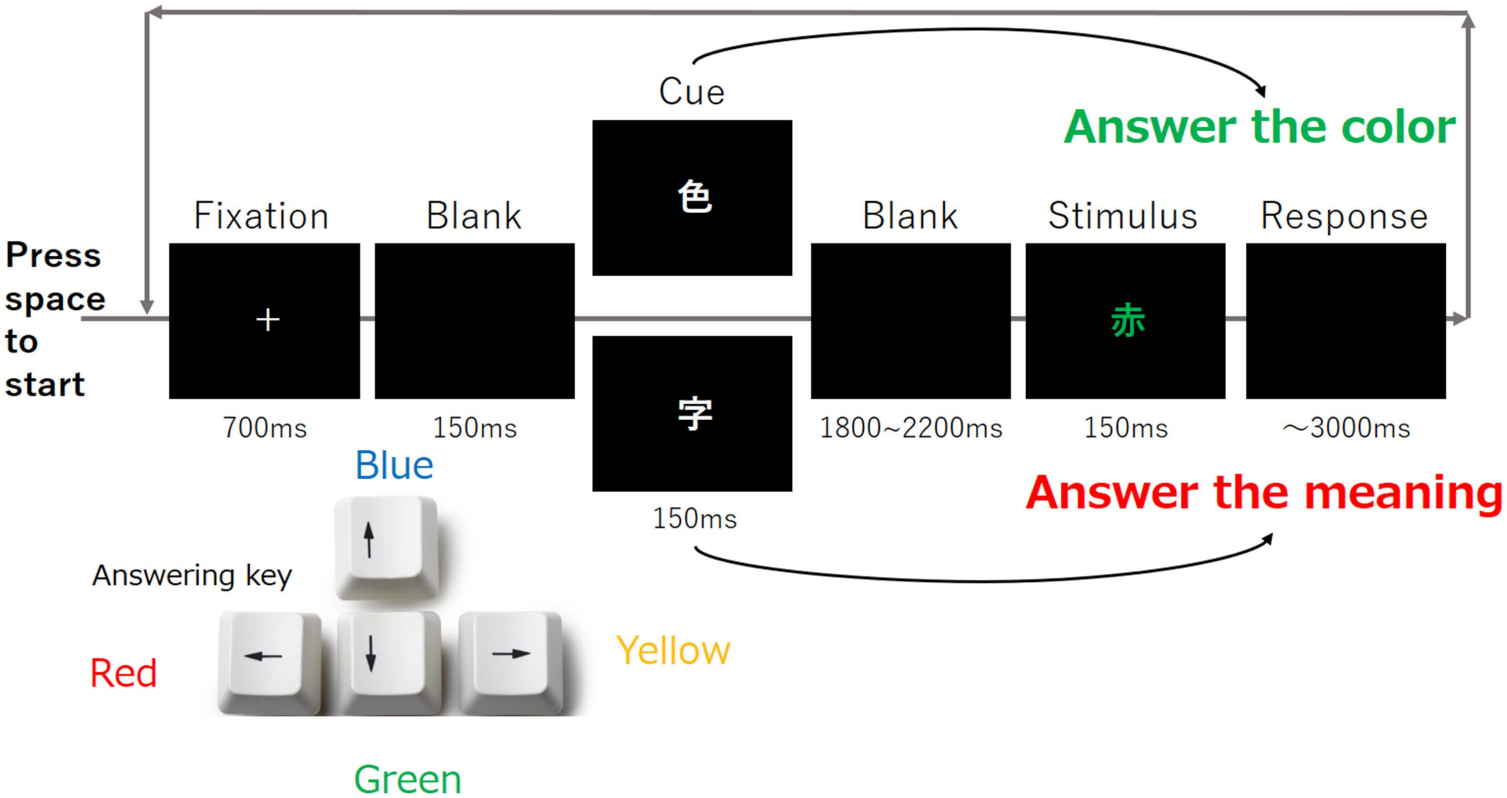
Overview of the Stroop task. Each trial was progressed based on this figure, and each participant was instructed to respond to the color of the stimulus by pressing the response key shown in the lower left corner of the figure.

The Stroop task was performed as follows: Each participant was asked to open his/her eyes and perform the Stroop task, as shown in Figure 4. In one task, the fixation point was first displayed for 700 ms, followed by 150 ms of blank, 150 ms of cue, 1,800–2,200 ms of blank, and 150 ms of stimulus. The participant was required to press the corresponding key within 3,000 ms.

The Stroop task comprised two types of cues and a series of stimuli. The cue was composed of the kanji “色” and another kanji “字.” For the cue, only one kanji character either “色” or “字” was presented. When participants saw the cue represented by “色,” they were asked to identify the color of the next kanji stimulus. When participants saw the cue represented by “字,” they were asked the meaning of the next kanji stimulus. The duration of the second blank was determined randomly as described in Soar et al. (2016). The participants were required to press the corresponding key as shown in Figure 4. This task was repeated 100 times using different stimuli.

#### 2.1.7 EEG recording

**Figure 5:**
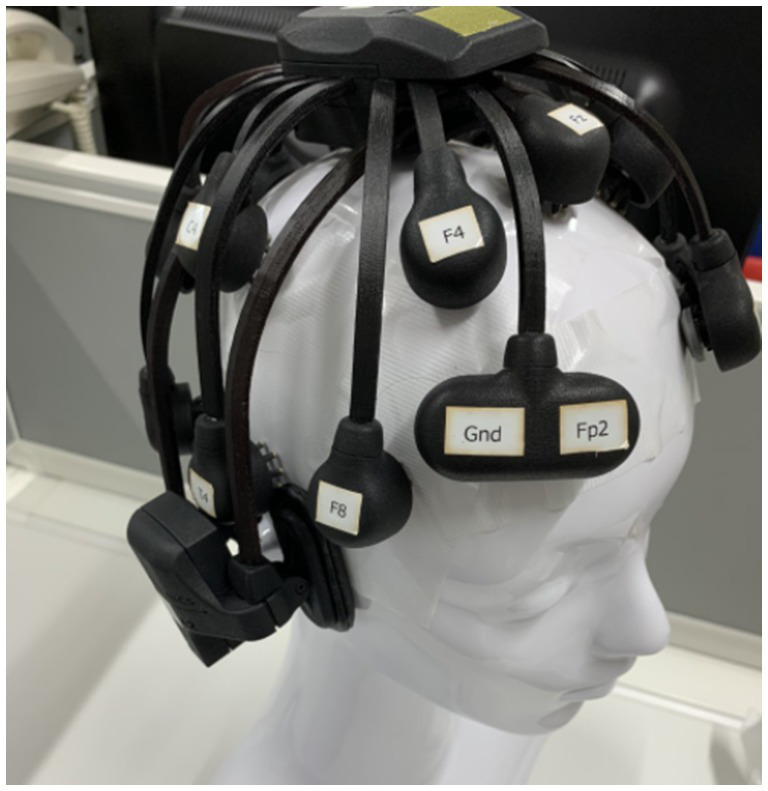
Mobile dry-EEG headset used in the experiment.

**Figure 6:**
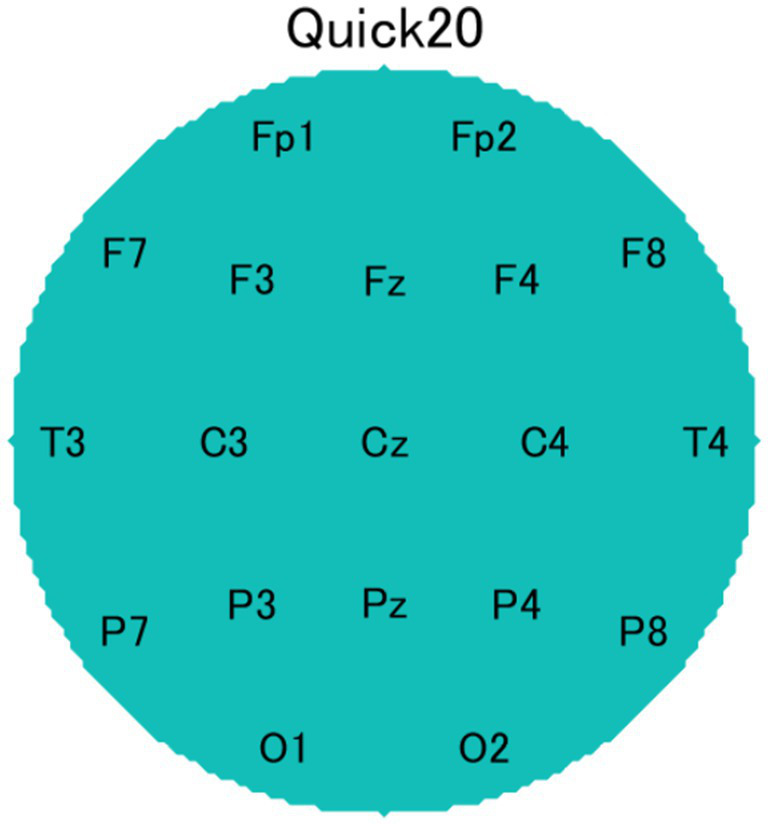
Electrode configuration based on the international 10–20 system.

The EEG data were recorded using Cognionics Quick-20 headsets (Figure 5, CGX, San Diego, CA, USA) consisting of 20 active dry electrodes (Figure 6). These electrodes were placed according to the international 10–20 system and the recorded signals were digitized at a sampling rate of 500 Hz. The reference electrode was placed at A1, and the ground electrodes were placed near Fp1 and Fp2. An additional electrode (A2) was placed for measurement. Impedance levels were kept below 500 kΩhm. EEG data were recorded using Data Acquisition Software (CGX, San Diego, CA, USA).

### 2.2 Data analyses

#### 2.2.1 Behavior data analyses

We used two methods to analyze participant behavior: (1) correct answer rate on the Stroop task for all four blocks and (2) the total working time for each Stroop task was measured from the beginning of the task to the 100th response. Furthermore, regarding unanswered trials, in the analysis of accuracy, these trials were treated as incorrect responses. In the analysis of working time, since participants were required to respond within 3 s, any trial exceeding this time limit was treated as having a response time of exactly 3 s.

#### 2.2.2 EEG analyses

The raw EEG data were processed using a notch filter at 60 Hz. The recorded signals were referenced to the average values of A1 and A2. All data were transformed into frequencies using the discrete Fourier transform method with the fft (x) function in MATLAB (MathWorks, Inc., Natick, MA, USA), and the spectral amplitude (μV) of the data was computed. The mean amplitudes of each EEG band were calculated as follows: theta (4 ≤ theta band < 8 Hz), alpha (8 ≤ alpha band < 14 Hz), beta (14 ≤ beta band < 30 Hz), and gamma (30 ≤ gamma band < 50 Hz). For representative values, the median of 100 mean amplitudes was calculated for each band at each electrode. For further analyses, the relative EEG activity was evaluated using decibels (dB) based on block 0.

#### 2.2.3 EEG lateralization analyses

This study also focused on the participants’ motivation. We defined three laterality indices of alpha band activity in the three cortical regions.

- Frontal region: (*F4* + *F8*)/2 − (*F3* + *F7*)/2
- Temporal region: (*T4* + *C4*)/2 − (*T3* + *C3*)/2
- Parietal region: (*P4* + *P8*)/2 − (*P7* + *P3*)/2

Here, each name represents the relative amplitude of the electrode (dB).

#### 2.2.4 Statistical analyses

EEG data, behavior, and questionnaires were statistically analyzed by EZR (Saitama Medical Center, Jichi Medical University, Saitama, Japan), which is a graphical user interface for R (The R Foundation for Statistical Computing, Vienna, Austria); and Bell Curve for Excel (Social Survey Research Information Co., Ltd., Tokyo, Japan) as follows:

- To investigate the difference in correct answer rate and working time on Stroop tasks between groups 1 and 2, a repeated measures analysis of variance was performed.
- A scatter plot was drawn to visually check for a normal distribution. As a result, much of the EEG data did not appear to be normally distributed; therefore, the Mann–Whitney U-test was performed. We used the U-test to compare each electrode in the two conditions and to compare the laterality in the brain.
- The Bonferroni correction was used for repetition of statistical tests.

### 2.3 Results

#### 2.3.1 Questionnaire

In the questionnaire used to confirm the participants’ current state, there were four questions: “satiety,” “fatigue,” “fried rice deliciousness,” and “arousal level.” However, none of the four questions showed significant differences between the groups or blocks.

#### 2.3.2 Behavior

**Figure 7:**
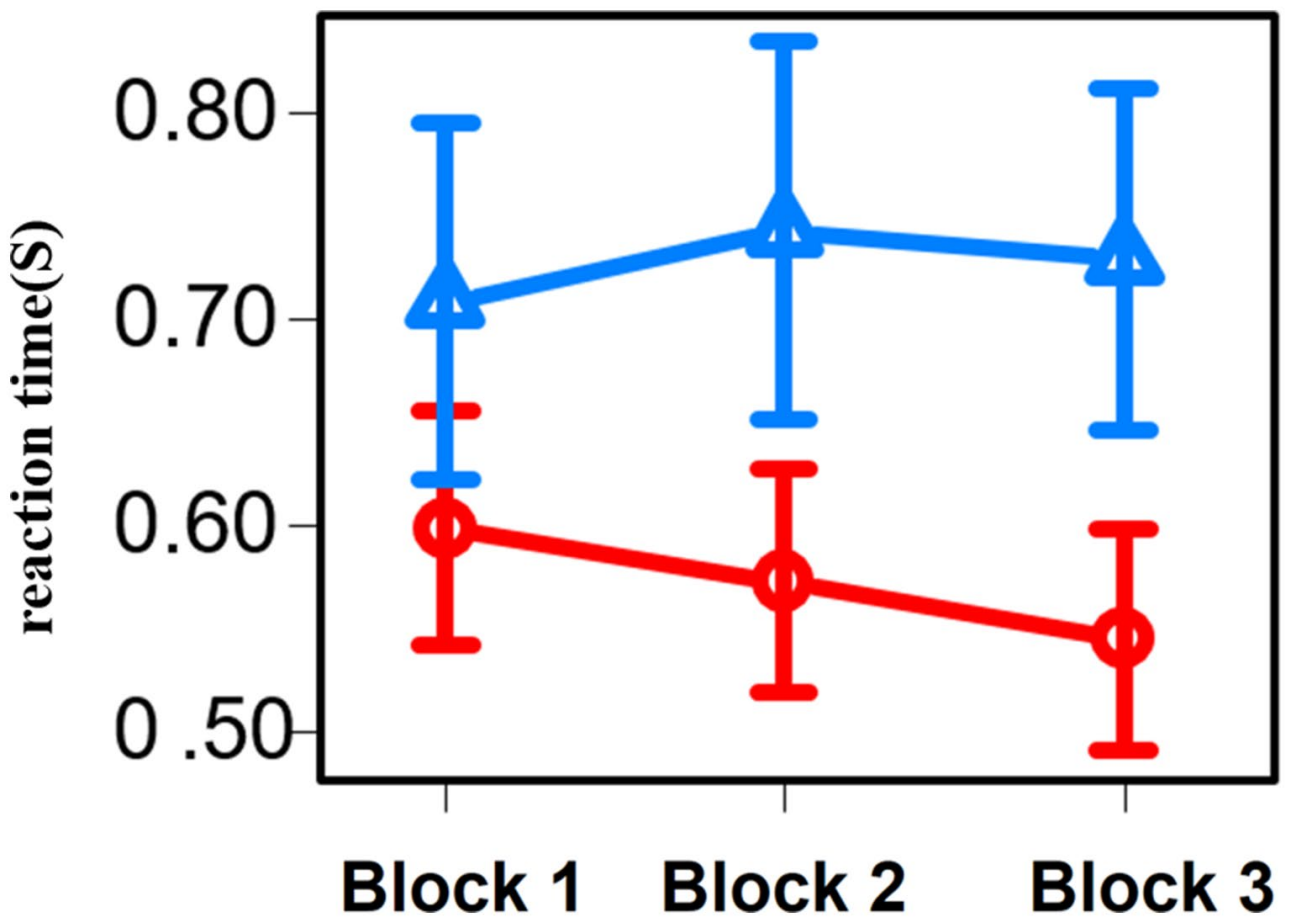
Line graph of the reaction time for each three blocks of the Stroop task. Red represents the results of group 1 and blue represents the results of group 2.

To understand how the different questions affected the behavior of participants, we analyzed the correct answer rate and reaction time for each of the three blocks of the Stroop task separately between groups 1 and 2 (Figure 7).

For the correct answer rate across all blocks of the Stroop task, there were no significant differences between the participants in groups 1 and 2 (*p* = 0.86). Regarding the working time across all block of the Stroop task, group 1 was significantly lower than group 2 (*p* = 0.01).

#### 2.3.3 EEG

To investigate the relationship between food deliciousness and EEG, we analyzed the theta, alpha, beta, and gamma amplitudes at all electrodes under two conditions (groups 1 and 2). For the Stroop task, we created a topography for each block to compare the overall results of groups 1 and 2. Different spatial distributions were observed in all bands for each block.

##### 2.3.3.1 Block 1

**Figure 8:**
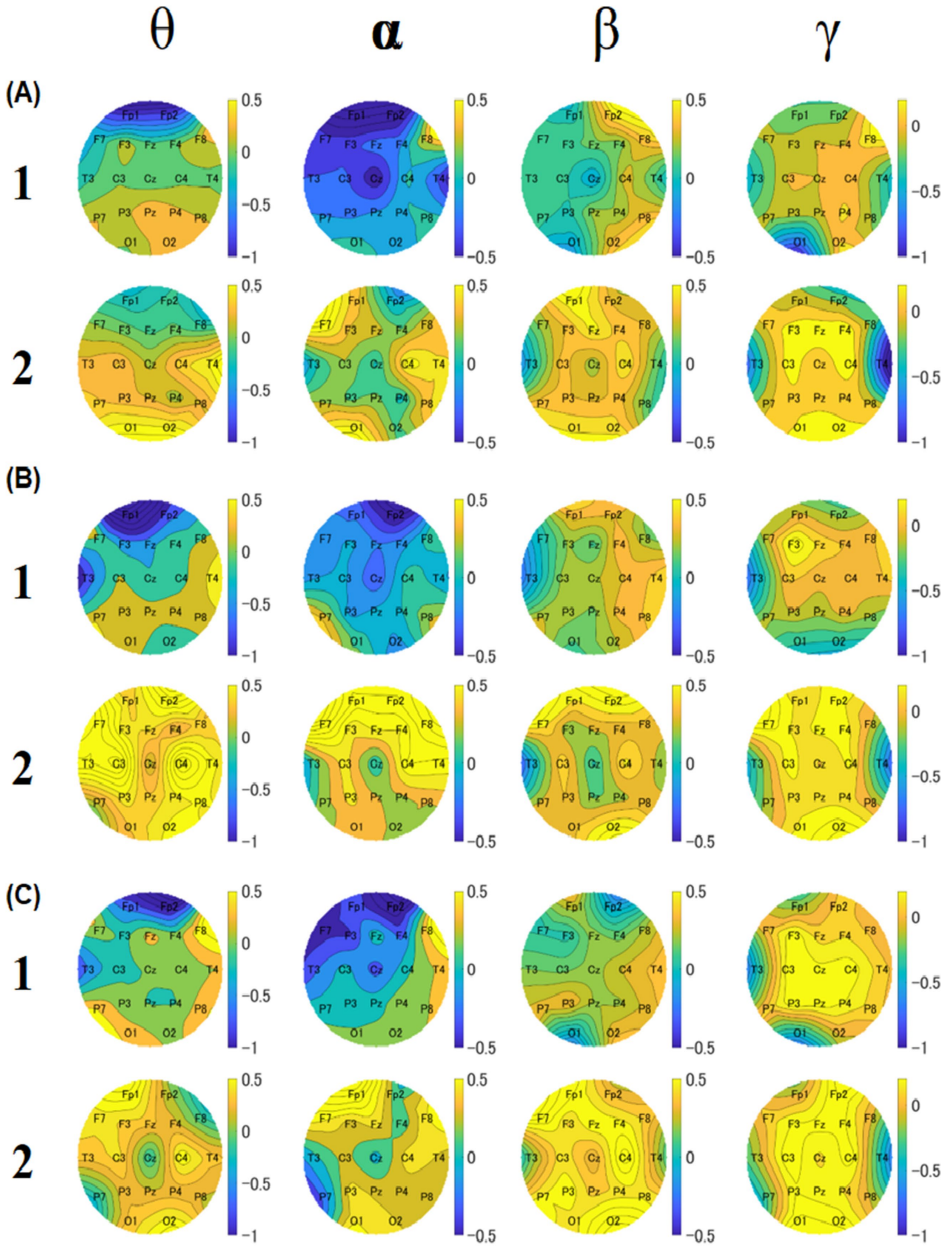
Topographies of median values of relative amplitudes for block 1 **(A)**, 2 **(B)**, and 3 **(C)**. For each block, the upper row shows the results of group 1, and the lower row shows the results of group 2. The four columns show theta, alpha, beta, and gamma results, respectively. The color bar represents the relative amplitude (dB).

In block 1, the theta, alpha, and beta amplitudes of most regions in group 1 were lower than those in group 2 (Figure 8). The amplitude of theta band was low near the frontal lobes in both groups. The overall amplitudes of the alpha band in group 1 were lower than those in group 2. The amplitudes of the beta band near the left parietal lobes and Cz of group 1 were lower than those of the other electrodes of group 1 and lower than those of the whole region in group 2 in block 1 and block 3. The amplitudes of the gamma band near the left occipital area in group 1 were lower than those in the other regions. The amplitudes near the right temporal area of group 2 were lower than those of the other regions.

**Figure 9:**
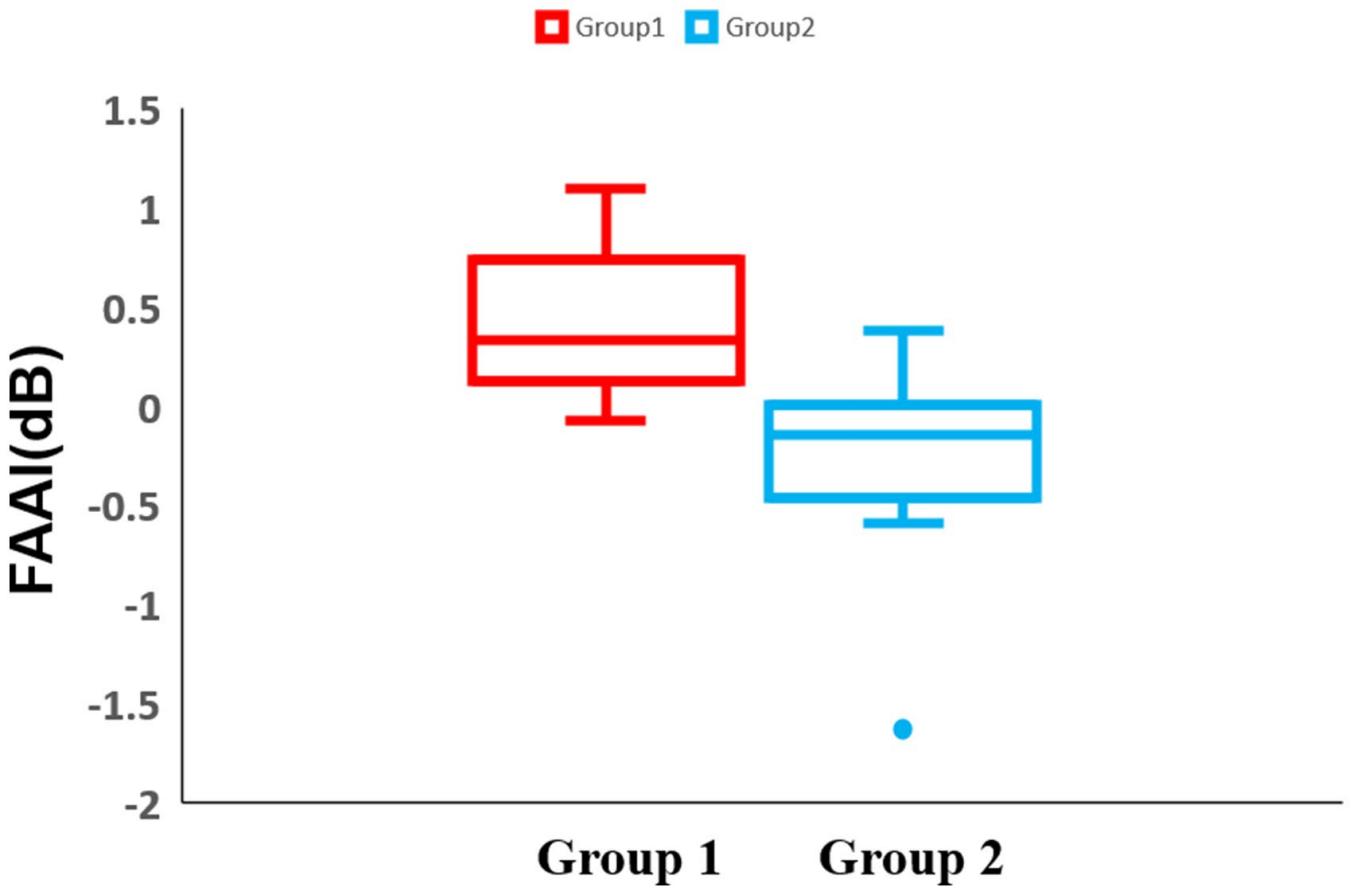
Box-and-whisker diagram of the lateralization analysis in block 1. The horizontal axis of the table represents two distinct experimental groups, while the vertical axis denotes the Frontal Alpha Asymmetry Index (FAAI). The middle line represents the median. Red represents the results of group 1 and blue represents the results of group 2.

To make the motivation easier to understand, a lateralization analysis was performed across all blocks. In the frontal region of block 1, there was no significant difference between the two groups 1 and 2 (Group 1: median = 0.33 dB, IQR = 0.61 dB, Group 2: median = −0.15 dB, IQR = 0.47 dB, *p* = 0.0028) (Figure 9). For the temporal and parietal regions, there were no significant differences between groups 1 and 2.

##### 2.3.3.2 Block 2

In block 2, the theta, alpha, and gamma amplitudes of most electrodes in group 1 were lower than those in group 2 (Figure 8). The amplitudes of the theta band near the left temporal and frontal areas in group 1 were lower than those in group 2. The overall amplitudes of the alpha band in group 1 were lower than those in group 2. As for the beta band, it was found that the amplitudes near the right temporal and parietal lobe areas of group 1 were lower than those of the other regions of group 2 and lower than those of the whole region of group 2. Regarding the gamma band, the amplitudes near the left temporal and occipital lobe areas of group 1 were lower than those in the other regions. The amplitudes near the temporal region were lower in group 2 than in other regions.

Regarding the lateralization analysis in block 2, there were no significant differences between groups 1 and 2 for all regions.

##### 2.3.3.3 Block 3

In block 3, the theta, alpha, and beta amplitudes of most regions in group 1 were lower than those in group 2 (Figure 8). The theta band amplitudes near the left temporal and frontal areas in group 1 were lower than those in group 2. The overall amplitudes of the alpha band in group 1 were lower than those in group 2. As for the beta band, the amplitudes near the near left temporal, right frontal, and left occipital areas near the left temporal, right frontal, and left occipital areas of group 1 were lower than those of the other electrodes of group 2 and lower than the whole region of group 2. For the gamma band, the amplitudes in the left temporal and left occipital areas in group 1 were lower than those in the other regions. The amplitudes near the temporal region in group 2 were lower than in the other regions.

**Figure 10:**
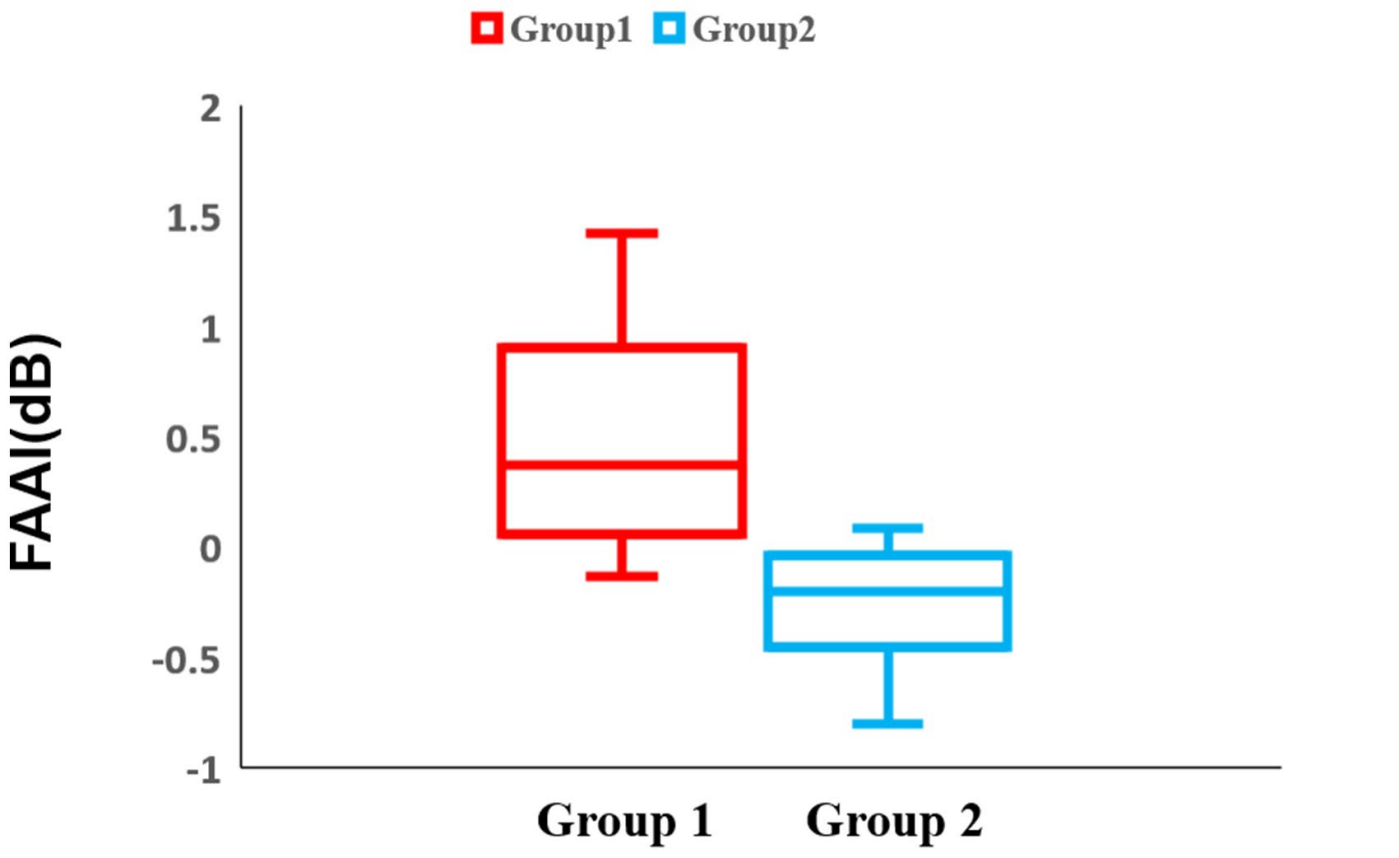
Box-and-whisker diagram of the lateralization analysis in block 3. The middle line represents the median. Red represents the results of group 1 and blue represents the results of group 2.

Regarding the lateralization analysis in block 3, there was a significant difference in the frontal region between groups 1 and 2 (Group 1: median = 0.367 dB, IQR = 0.84 dB, Group 2: median = −0.20 dB, IQR = 0.41 dB, *p* = 0.0014) (Figure 10). For the temporal and parietal regions, there were no significant differences between groups 1 and 2.

##### 2.3.3.4 *P*-value topography

**Figure 11:**
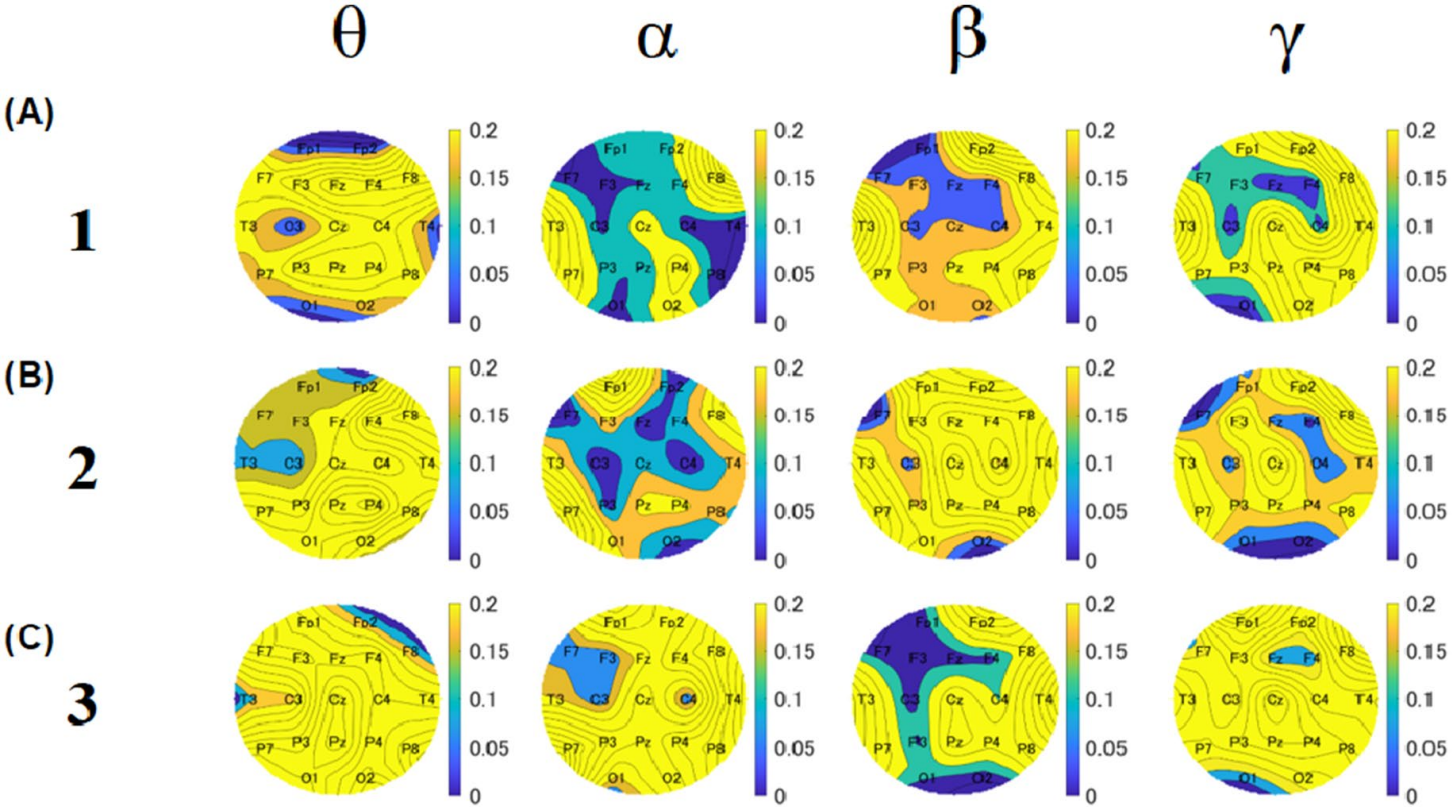
Topographies of *p*-values based on U-test between group 1 and group 2. The upper, middle, and lower rows show the results of blocks 1 **(A)**, 2 **(B)**, and 3 **(C)**. Five columns show theta, alpha, beta, and gamma results, respectively. The color bar represents the relative amplitude (dB).

To observe the overall EEG differences and explore the local EEG differences, we created *p*-value topographies based on the Mann–Whitney U-test of all electrodes for participants between groups 1 and 2 (Figure 11). These results were obtained without Bonferroni correction and will only be used for discussion, references, and future research directions. We focused on the common points in all three blocks. As for the theta band, the *p*-value for C3 was lower for both blocks 1 and 2. For the alpha band, the *p*-values for C3, C4, and F7 were lower than those of the other electrodes across all blocks. For the beta band, the *p*-value for C3 was the lowest across all blocks. For the gamma band, the *p*-values for Fz and C4 were lower across all blocks.

## 3 Experiment 2

### 3.1 Materials and methods

#### 3.1.1 Participants

Twenty-nine students (12 females, no history of neuropsychiatric illness or hearing impairment, and normal hearing in both ears) participated in this study. One participant withdrew from the experiment due to poor physical health unrelated to the experiment. Two other participants were excluded from the analysis due to errors in the data resulting from equipment malfunction. Only 26 participants were included in this experiment. The mean age of the patients was 23 years (range, 19–28 years; SD: 2.6). All participants were recruited from Kyushu University, Japan. All participants had normal or corrected-to-normal vision. The participants were requested to complete their meal at least 2 h prior to the start of the experiment and to remain hungry during participation.

This experiment started on 10/04/2023, and ended at 28/10/2023. As in Experiment 1, Kyushu University Research Ethics Committee, Reference number 202104, also approved Experiment 2 and the participants consented to participate in the informed consent process. Participants who had been involved in Experiment 1 were excluded from participation in Experiment 2. Consequently, all individuals recruited for Experiment 2 were naïve to the experimental protocol and consisted of students with no prior exposure to similar cognitive paradigms.

#### 3.1.2 Food samples

In this experiment, sample 1 of the fried rice was selected based on the highest average score of the preliminary experiment (7.6 out of 10); sample 2 was selected based on the second-highest average score (6.8 out of 10).

In Experiment 1, sample selection was based on deliciousness assessments conducted during preliminary testing; however, these evaluations were not administered to all participants. To address this limitation, in Experiment 2, deliciousness ratings were administered to all participants.

#### 3.1.3 Experiment design

In Experiment 1, only one type of sample was consumed by each participant, thus it is currently unclear what difference would occur when the participants compare two different types of samples. Consequently, in Experiment 2, we altered our methodology by dividing the two-week period into two distinct phases, with each participant consuming two samples. However, to account for order effects, a schedule was created in which half of the participants took sample 1 first in week 1 and the other half took sample 2 first in week 1.

#### 3.1.4 Correlation analyses

Due to the disparate schedule of Experiment 2, it was postulated that confounding variables might result in adverse outcomes. Consequently, samples 1 and 2 were merged for a Spearman’s rank correlation analysis of palatability and EEG.

### 3.2 Results

#### 3.2.1 Questionnaire

**Figure 12:**
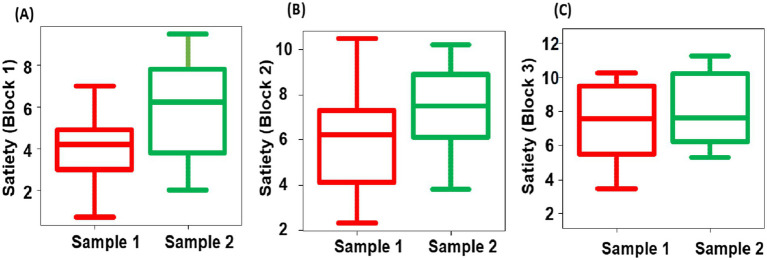
Box-and-whisker diagram of the Friedman’s test of satiety in block 1 **(A)**, 2 **(B)**, and 3 **(C)**. The middle line represents the median. Red represents the results of sample 1 and green represents the results of sample 2.

A four-question questionnaire was administered in the same manner as in Experiment 1: “satiety,” “fatigue,” “fried rice deliciousness,” and “arousal level.” For the Friedman’s test results on satiety level of the two types of fried rice, sample 1 exhibited significantly lower levels than sample 2 for blocks 1 (*p* = 0.02), 2 (*p* < 0.001) and 3 (*p* = 0.03) (Figure 12). However, the remaining three questions yielded no significant differences between the samples or blocks. For deliciousness ratings in Experiment 2, there were no significant difference between two groups (group 1: mean score = 7.5 ± 1.3 SEM; group 2: mean score = 7.2 ± 1.2 SEM).

#### 3.2.2 Behavior

The correct answer rate and reaction time for each of the three blocks of the Stroop task were analyzed separately between samples 1 and 2, as in Experiment 1. Unlike Experiment 1, there was no significant difference between the two samples in both correct response rate (block 1 *p* = 0.29, block 2 *p* = 0.94, block 3 *p* = 0.97) and working time (block 1 *p* = 0.53, block 2 *p* = 0.79, block 3 *p* = 0.83).

#### 3.2.3 EEG

##### 3.2.3.1 *P*-value topography

**Figure 13:**
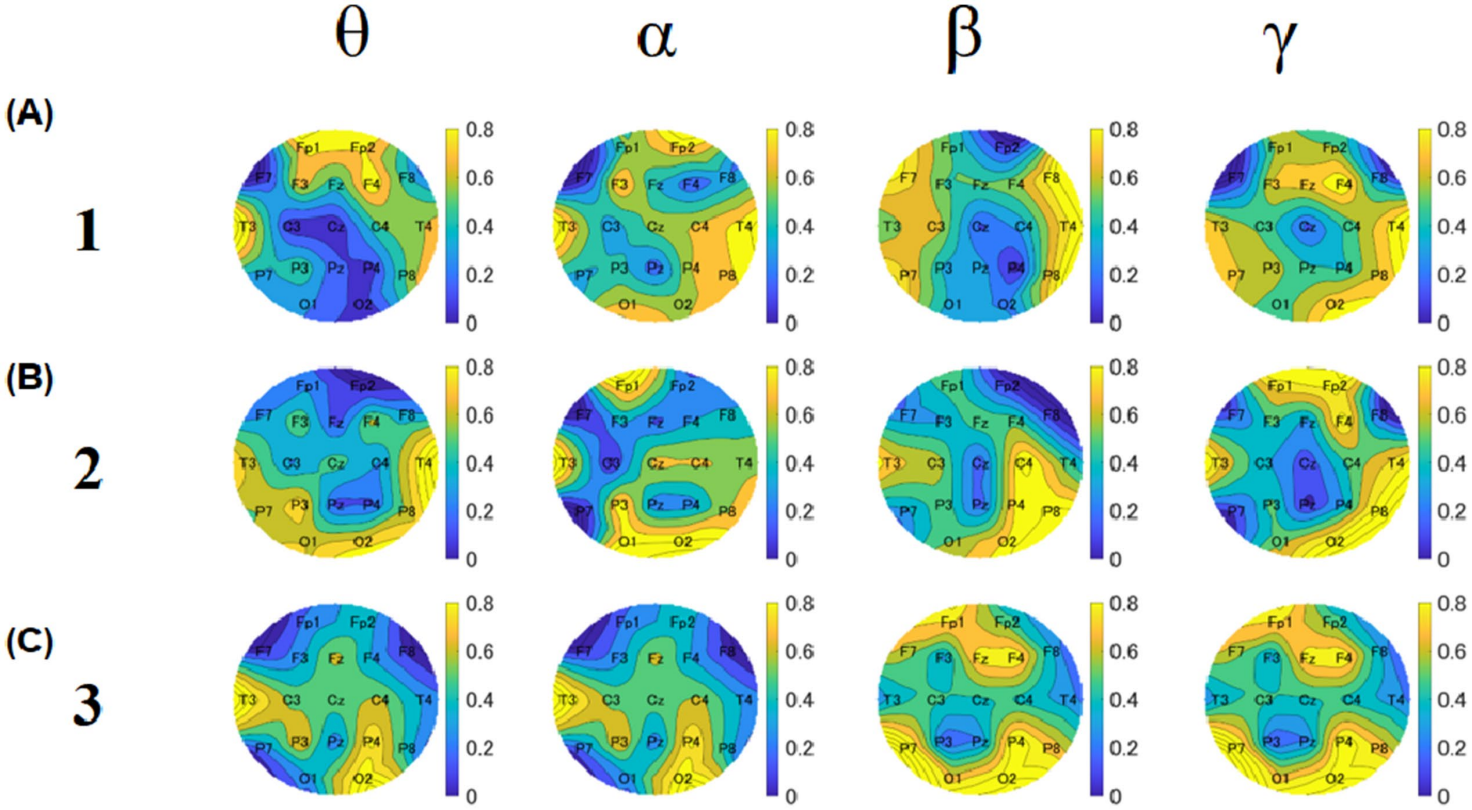
The topographies of *p*-values based on the Friedman’s test between sample 1 and sample 2. The upper, middle, and lower rows show the results of blocks 1 **(A)**, 2 **(B)**, and 3 **(C)**. Five columns show theta, alpha, beta, and gamma results, respectively. The color bar represents the relative amplitude (dB).

A Friedman test was conducted across 19 electrodes. However, after applying a correction for multiple comparisons using the Benjamini & Hochberg method, most of the significant differences were no longer present. Nevertheless, a trend toward significance remained at the C3 electrode in the theta band between block 1 (corrected *p* = 0.024) and block 2 (corrected *p* = 0.072). To observe overall EEG differences and explore local EEG differences, we conducted a Friedman’s test on each electrode for samples 1 and 2 and obtained a *p*-value for each electrode (Figure 13). To identify specific brain regions and electrodes for our future study, we created a *p*-value topography for Experiment 2. As for the theta band, the *p*-value for F7 was lower for both blocks 1 and 3. For the alpha band, the *p*-values for F7 and Pz were lower than those of the other electrodes across all blocks. For the beta band, the *p*-value for Fp2 was the lowest for blocks 1 and 2. For the gamma band, the *p*-values for F7 and F8 were lower across all blocks 1 and 2.

##### 3.2.3.2 Spearman’s rank correlation analysis

**Figure 14:**
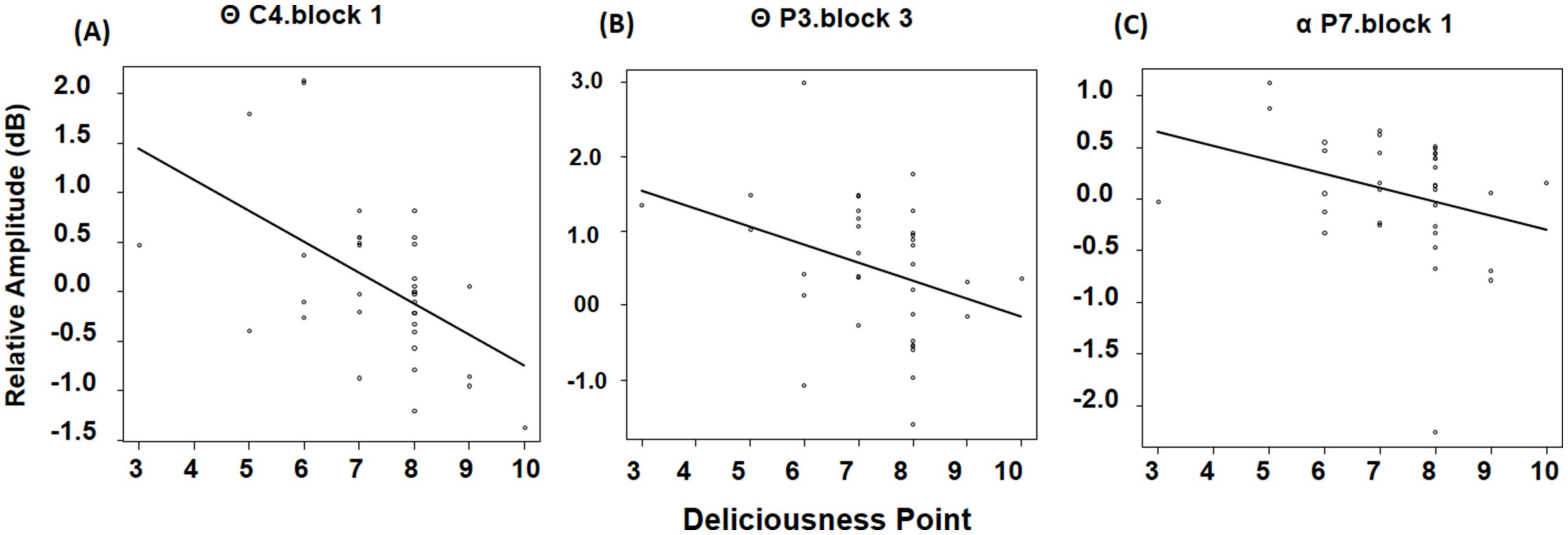
Spearman’s rank correlation of deliciousness and EEG for theta and alpha band. The vertical axis represents theta or alpha waves, while the horizontal axis depicts the scores for the tastiness scale. **(A)** The correlation result of C4 electrode for theta band in block 1. **(B)** The correlation result of P3 electrode for theta band in block 3. **(C)** The correlation result of P7 electrode for alpha band in block 1.

As for the theta band, deliciousness and EEG were negatively correlated at C4 electrodes (*p* < 0.001) for block 1 (Figure 14A). In block 3, deliciousness and EEG were negatively correlated at the P3 electrode (*p* = 0.02) (Figure 14B). For the alpha band, deliciousness and EEG were negatively correlated at the P7 electrodes (*p* = 0.05) for block 1 (Figure 14C).

## 4 General discussion

### 4.1 Questionnaire

In Experiment 1, the questionnaire was primarily a subjective evaluation, and as there were 20 participants, large individual differences could exist; it would be difficult to find significant differences between the two groups. The difference in brain activity may not have been noticed by the participants themselves; therefore, we suggest that the subjective evaluation did not show any difference.

Experiment 2 demonstrated that satiety in sample 2 was significantly higher than in sample 1 in block 1 and block 2. A previous study (Chaput and Tremblay, 2007) reported that a cognitive task before eating also influenced satiety levels. The results indicate that the more challenging the task, the stronger the feeling of hunger. However, in Experiment 2, all participants performed the same task, and the stimulus presentation was random, so the effect of task difficulty could be considered negligible. Conversely, there have been numerous studies on food taste, appetite, and satiety levels. For instance, Mendoza et al. (2005) proposed that an elevation in motivational state resulting from the palatability and nutritional value of a meal is sufficient to induce a specific behavioral response. Bernard’s study (Bernard et al., 1994) demonstrated that energy intake was influenced by the level of deliciousness of a conventional meal. Consequently, the findings of Experiment 2 indicate that all participants were less likely to feel satiated due to the superior taste of Sample 1 compared to Sample 2.

### 4.2 Behavior

#### 4.2.1 Working time

In Experiment 1, the working time on the Stroop task for the participants in group 1 was shorter than that in group 2. There are several possible explanations for these results. First, Yau and Potenza (2013) proposed that activity in some brain regions is higher during a preferred attentional focus. Sample 1 was found to be rated as the most delicious in our preliminary experiment and had the best taste in this experiment. We suggest that after eating more delicious food, participants concentrated more on the Stroop task; therefore, the entire working time of the task was shorter than that of group 2. Second, previous studies have reported that an individual’s level of arousal is related to food intake. Stroebele and De Castro (2004) recruited college students to conduct experiments that examined the relationship between physiological factors such as arousal and food. Therefore, we suggest that eating more delicious food made participants more efficient in the Stroop task.

#### 4.2.2 Correct answer rate

For the correct answer rate in Experiment 1, we found that the percentages of correct responses in all blocks were high. There were no significant differences between groups 1 and 2 in any of the blocks.

The probable reason can be considered as follows: first, all the participants in this experiment were university students and therefore could easily become accustomed to the task of this experiment. Second, Huseynov and Palma (2021) showed that participants obtained information with time pressure as effectively as without it. In this experiment, the response time of one trial was set within 3,000 ms; however similar to the results of Huseynov and Palma (2021), the limited response time did not seem to have a significant impact.

### 4.3 EEG

#### 4.3.1 Alpha

It is known that the greater the decrease in the alpha amplitude, the more aroused the participants become. Klimesch et al. (2007) that the alpha band plays a role in inhibition and timing and correlates with cognitive effects and states of excitation and arousal. As we introduced a cognitive task in this study, we focused more on the alpha band. The relationship between arousal and the alpha band was also studied by Ajjimaporn et al. (2022) using caffeine in college students. These results support the arousal effects of caffeine and suggest a decrease in the resting alpha wave activity after caffeine consumption. In our study, the alpha amplitude of group 1 was lower than that of group 2 in all cortical regions in all blocks. This result indicates that the participants in group 1 were more aroused than those in group 2 during the Stroop task. Our preliminary experiments showed that sample 1 was the most delicious, and this result also indicates that participants can become more aroused after eating more delicious food.

In a study of wine, with the sedating effect of wine on participants, the participants’ alpha amplitudes increased, indicating a lower arousal level. In this experiment, the participants in group 2 showed a relatively high alpha amplitude. This may indicate a lower level of arousal, as shown in the results of the Sanz-Martin et al. (2011). Jensen and Tesche (2002) analyzed EEG signals during the retention interval of a modified Sternberg task. They showed that alpha amplitude increased with memory load in simple working memory tasks. Our study also indicated that the alpha amplitudes of group 1 were lower, suggesting that eating delicious foods would result in higher work efficiency.

Lateralization analysis is frequently used in studies on approach and withdrawal motivation (Harmony et al., 1999). There have been many studies on FAA (Harmon-Jones et al., 2010; Allen and Reznik, 2015). In a study by Kirsten et al. (2022), participants were asked to perform a food-choice task and were provided with two food choices: one high-calorie and one low-calorie.

Analysis of the EEG results revealed that higher alpha asymmetry was associated with stronger activity in the left frontal region in response to low-calorie food and stronger approach motivation. In this study, we developed a lateralization index that considers large individual differences instead of FAA. The lateralization analysis results showed that group 1 had significantly higher values than group 2. This indicates that the left frontal region was more active in group 1. Similar to the results of Kirsten et al. (2022), that is, the left frontal region activity is related to approach motivation, our results also indicate that participants showed approach motivation after eating more delicious food. No significant differences were observed in the lateralization analyses of the temporal and parietal regions. This is also consistent with the fact that previous studies have failed to find significant results for this region.

#### 4.3.2 Theta

Cavanagh and Frank (2014) showed that the theta band activity in the frontal cortex reflects the general calculations used to recognize the need for cognitive control. Gevins et al. (1997) performed four versions of a continuous matching task and recorded participants’ EEG. They showed that the theta power increased during difficult experimental tasks, such as increases in stimulus complexity. Harmony et al. (1999) also reported that task load affected the theta waves of participants. They used narrowband analyses to examine the EEG in calculation and control tasks. They showed that an increase in the theta amplitude presumably corresponded to a higher task load. In Experiment 1, the theta amplitudes of group 1 were lower than those of group 2 in all blocks. This may indicate that the brain activity of group 1 was in a lower load condition after eating more delicious food.

There are many studies on the increase in theta band power (Cohen and Donner, 2013). Furthermore, it was reported that increased frontal theta power was associated with impaired inhibition, working memory, and planning (Wall et al., 2022). It has also been reported that frontal theta neurons may undergo emergent processes such as cognitive control (Cavanagh and Frank, 2014). Cohen and Donner (2013) analyzed EEG signals during visual and auditory tasks. They reported that neural reactions to conflict, punishment, and error manifestations were positively correlated with increases in the theta amplitude. In Experiment 1, we suggest that there could be more conflicting and more negative responses in group 2 than in group 1 when responding to the Stroop task; hence, the theta amplitudes of group 2 were higher than those of group 1.

#### 4.3.3 *P*-value topography

In Experiment 1, the most significant differences between groups were in C3 and C4, which are closer to the somatosensory cortex. According to the *p*-value results, group 1 exhibited lower alpha amplitudes at the C3 and C4 electrodes than group 2 (see C3 and C4 of the alpha in Figure 11). For the theta band, a relatively low *p*-value was observed in the C3 electrodes between groups 1 and 2 (see C3 of theta in Figure 11). The theta amplitudes of the C3 electrode in group 1 were lower than those in group 2. The EEG activity at C3 primarily represents sensory-motor cortical activity. Theta band activity at C3 has been reported to increase when cortical excitability is reduced. These results indicated that consuming delicious food activates the somatosensory cortex. Previous studies have shown that the somatosensory cortex modulates motor control by facilitating or inhibiting inappropriate information (Wasaka and Kakigi, 2012). It was also indicated that alpha ERS plays a direct and active role in somatosensory perception and attention in the SII (Dockstader et al., 2010).

The Wernicke’s area has been reported to be related to word recognition (DeWitt and Rauschecker, 2013). In our study, C3 electrodes were close to Wernicke’s area, and the alpha amplitude in C3 electrodes was low in group 1. In addition, participants understood the meaning of the kanji characters during the Stroop task; it is thought that Wernicke’s area was also active after eating delicious food. The left Wernicke’s areas function as processors of dominant meanings of ambiguous words (Harpaz et al., 2009). In this study, group 1’s Wernicke’s areas were more active, suggesting that after eating more delicious food, group 1 performed the Stroop task more seriously and intensively than group 2.

Gamma band activity is also often studied in relation to emotions. High levels of gamma activity at posterior electrode sites have been reported to be associated with negative emotions (Green and Nachtigal, 2012). In the present study, the *p*-value topographies exhibited relatively low *p*-values near the posterior region. A comparison of the gamma amplitudes near the posterior region between the two groups showed that group 2 had a higher gamma amplitude. Therefore, we propose that people experience more negative emotions after eating normal food.

#### 4.3.4 Spearman’s rank correlation analysis

In Experiment 2, the EEG data analysis was unable to show any significant differences among the samples. This may have been caused by confounding factors, individual differences in preferences among participants, and the order in which the samples were eaten. However, a correlation analysis of the overall taste and EEG data demonstrated some significant differences without dividing the samples. A significant difference was observed in theta and alpha waves between the two groups in block 1. The study comprised four blocks, with block 0 representing a baseline period during which participants appeared to be unfamiliar with the task, anxious, and affected by hunger. In block 1, participants were considered to be in the optimal state for eating evaluation, as they exhibited a lower level of fatigue and were more familiar with the task.

Previous studies have also demonstrated that theta activity increases with working memory load, suggesting a potential role for theta oscillations in the maintenance of working memory (Gevins et al., 1997). The results of Experiment 2 showed a negative correlation between the palatability of the sample and the theta power of Fp1 electrode. This phenomenon may imply that the consumption of a delicious food can result in a reduction in the cognitive load associated with memory. Previous research has indicated a correlation between theta activity and forecasting abilities (Cavanagh and Frank, 2014). Crivelli’s study (Crivelli-Decker et al., 2018) indicated that theta power diminishes as an individual’s forecasting proficiency increases. The results of Experiment 2 also indicate that after eating a delicious meal, participants demonstrated enhanced proficiency in the task and exhibited active anticipation of the subsequent stimulus. For alpha band, previous research (Klimesch, 1999) had indicated that the degree of alpha power suppression was positively correlated with cognitive ability. In Experiment 2, higher scores for the subjective experience of deliciousness were found to be associated with a suppression of alpha power. The results indicate that the cognitive performance of the participants was enhanced following the consumption of the sample, which was perceived as delicious. The findings of Klimesch et al. (2007) indicated that a reduction in alpha power is associated with increased arousal. The results of Experiment 2 are consistent with those of Experiment 1, demonstrating that participants exhibited heightened arousal following the consumption of the perceived delicious sample.

### 4.4 Limitations

In Experiment 1, a total of 25 participants were recruited during the COVID-19 pandemic. Five participants were excluded and the remaining participants were divided into two groups of 10 each. This grouping resulted in a small number of participants and large individual differences. In addition, all participants were university students. Because all participants had relatively similar body shapes, we did not examine their body mass index in detail, which may be considered that deliciousness evaluation can change significantly depending on body mass index, so it will be a limitation of this study. Another limitation is that the only food sample used in the experiment was fried rice; therefore, the conclusions of this study may not be generalizable to other foods. In addition, we only recorded EEG data and did not record event-related (De) synchronization and event-related potential. Therefore, we did not perform source estimation. Moreover, the design of the Stroop task may be too simple for participants, and more complicated tasks should be used. Direct studies on the neural representation of food deliciousness under different attentional states are limited; however, research in cognitive neuroscience has demonstrated that divided attention can modulate sensory processing (Pessoa et al., 2003). For example, studies on attentional control suggest that adding a cognitive task during eating may reduce neural activation in taste-related regions, such as the front parietal network (Corbetta et al., 2000) and the orbitofrontal cortex (Kastner et al., 1999). Once the technology to remove myoelectricity is established, EEG can be measured during eating, and brain activity related to taste can be examined. Future studies are necessary to recruit more participants from different age groups, examine their body mass index, obtain additional data, perform this experiment with other types of food samples, and ask participants to perform more complicated tasks to overcome these limitations. In addition, in future research, efforts should be made to reduce individual variability by increasing the number of measurements and averaging the results, among other possible improvements.

## 5 Conclusion

In this study, we recorded and analyzed EEG data following delicious food consumption by having participants perform a Stroop task. After eating delicious food, alpha and theta band activities decreased in the frontal brain region, reflecting higher arousal; alpha band activity decreased more in the left frontal brain region than in the right, reflecting higher motivation and work efficiency. This discovery highlights how food-induced brain changes may directly impact mental performance and goal-driven behavior. By identifying these neural patterns, our study opens new possibilities for designing diets tailored to improve focus in high-demand professions and offers a physiological basis for understanding food-related motivation.

## Data Availability

The raw data supporting the conclusions of this article will be made available by the authors without undue reservation.
